# E-Health Tools to Improve Antibiotic Use and Resistances: A Systematic Review

**DOI:** 10.3390/antibiotics9080505

**Published:** 2020-08-12

**Authors:** Érico Carvalho, Marta Estrela, Maruxa Zapata-Cachafeiro, Adolfo Figueiras, Fátima Roque, Maria Teresa Herdeiro

**Affiliations:** 1 iBiMED–Institute of Biomedicine, Department of Medical Sciences, University of Aveiro, 3800 Aveiro, Portugal; ericocarvalho@ua.pt (É.C.); mestrela@ua.pt (M.E.); 2 Department of Preventive Medicine and Public Health, University of Santiago de Compostela, 15702 Santiago de Compostela, Spain; maruxa.zapata@usc.es (M.Z.-C.); adolfo.figueiras@usc.es (A.F.); 3 Consortium for Biomedical Research in Epidemiology and Public Health (CIBER Epidemiology and Public Health-CIBERESP), 28001 Madrid, Spain; 4 Health Research Institute of Santiago de Compostela (IDIS), 15706 Santiago de Compostela, Spain; 5 Research Unit for Inland Development-Polytechnic of Guarda (UDI-IPG), 6300 Guarda, Portugal; froque@ipg.pt; 6 Health Sciences Research Centre, University of Beira Interior (CICS-UBI), 6200 Covilhã, Portugal

**Keywords:** clinical decision support system, CDSS, antimicrobial management, e-health

## Abstract

(1) Background: e-Health tools, especially in the form of clinical decision support systems (CDSSs), have been emerging more quickly than ever before. The main objective of this systematic review is to assess the influence of these tools on antibiotic use for respiratory tract infections. (2) Methods: The scientific databases, MEDLINE-PubMed and EMBASE, were searched. The search was conducted by two independent researchers. The search strategy was mainly designed to identify relevant studies on the effectiveness of CDSSs in improving antibiotic use, as a primary outcome, and on the acceptability and usability of CDSSs, as a secondary outcome. (3) Results: After the selection, 22 articles were included. The outcomes were grouped either into antibiotics prescription practices or adherence to guidelines concerning antibiotics prescription. Overall, 15 out of the 22 studies had statistically significant outcomes related to the interventions. (4) Conclusions: Overall, the results show a positive impact on the prescription and conscientious use of antibiotics for respiratory tract infections, both with respect to patients and prescribing healthcare professionals. CDSSs have been shown to have great potential as powerful tools for improving both clinical care and patient outcomes.

## 1. Introduction

After the appearance of antibiotics, common infections that previously caused death or illness started to be effectively treated [1]. However, in recent years, medical science has been challenged with the emergence of highly resistant bacterial strains dispersed across the world. This emergence is the result of several factors, such as worldwide travel activity and, especially, antibiotic misuse and overuse [2,3]. In the context of primary care, both antibiotic overuse and a lack of adherence to guidelines are prevalent, albeit underestimated, issues [4]. It has also been revealed that 20–50% of the total amount of antibiotics prescribed in intensive care are unnecessary or inappropriate [5].

Antibiotic overuse in respiratory tract infections is very evident [4]. Respiratory diseases, namely infections of the respiratory tract, are one of the leading causes of death and disability in the world and have been shown to have a high incidence [6,7]. These infections include common cold, pharyngitis, epiglottitis, and pneumonia. While symptomatic treatment is used for most viral infections, bacterial pneumonias are usually treated with antibiotics. However, while antibiotics are ineffective against viral pathogens [6] and should thus only be prescribed when secondary bacterial infections develop [8,9], a 2018 [4] study revealed that almost three-quarters of prescribed antibiotics were not prescribed according to guidelines, and only 11% of them were optimally prescribed.

While modern medicine constantly needs new types of antibiotics and antivirals to treat drug-resistant infections, the pipeline of new drugs is declining [10]. In order to compensate for the lack of new antibiotics, novel/innovative health tools may help to improve antibiotic use, with the aim of ultimately helping to control antimicrobial resistance. In the case of acute respiratory tract infections, probably the most impactful interventions involve the avoidance of the prescription of antibiotics for the treatment of conditions for which antibiotics use is not indicated. This is related to improving guideline adherence, since poor guideline adherence is one of the problems related to the inappropriate prescription of antibiotics [4,11].

Guideline adherence and healthcare quality can be improved using clinical decision support systems (CDSSs), which ultimately help to close the gap between optimal practice and actual clinical care. This is reflected in the reduction in medication errors [12,13]. Consider the fact that rules that are implicit in guidelines can be encoded into CDSSs, and clinical care pathways that have been shown to be difficult to implement in practices with low clinician adherence are now easier to put into practice [14]. CDSSs are information systems that directly aid in clinical decision-making about an individual, and are designed to improve clinical decision-making using multiple direct features, including guideline dissemination, alerts, reminders, drug dose calculations, reduction in antibiotics prescriptions, distribution, adequate consumption, and reduction in practice variation and error [13,15,16]. It also consists of software designed to be a direct aid in clinical decision-making. This software can allow information on a patient to be matched with a computerized database, and patient-specific recommendations can be presented to the clinician [17]. In more technically advanced systems, the characteristics of each patient are computerized, and the software algorithms generate specific recommendations.

Electronic health records (EHRs), computerized provider order entry systems (CPOEs), and CDSSs are powerful tools for providing a safer, more effective, and more efficient healthcare delivery [18]. That said, the use of information and communication technologies (ICTs) in health is transforming the provision and management of health care. ICT use can provide benefits not only in terms of obtaining health gains, but also in terms of monitoring, research, and demonstration, thus significantly contributing to the development of the knowledge and transparency of a system [19]. Health information technology (HIT) aims to improve the quality of care by optimizing the exchange and coordination of health care information, implementing state-of-the-art clinical practices and reducing medication errors and adverse events [12].

CDSSs can be seen as very useful tools for improving guideline adherence, particularly regarding antibiotic use for respiratory infections, especially when considering both the significant room for improvement and the high heterogeneity of clinical practices. With the increase in newly available information on antibiotic use, a multiplicity of modalities for educating and informing both patients and health professionals can be employed to supplement traditional educational methods [20]. The focus of this systematic review is to assess the influence of e-health tools, namely, CDSSs, on antibiotic use. Moreover, it will also evaluate the acceptability of e-health tools in relation to the prescription, dispensation and use of antibiotics by health professionals specifically for respiratory infections.

## 2. Materials and Methods

### 2.1. Protocol and Registration

We followed the guidelines in the PRISMA Statement [21] in conducting this systematic review and recorded this study in the international database of prospectively registered systematic reviews (PROSPERO) (reg. no. CRD42020167316) [22].

### 2.2. Search Strategy and Inclusion Criteria

For this systematic review, searches in the scientific databases, MEDLINE-PubMed and EMBASE, on 4 February and 21 February 2020, respectively, were conducted.

The search was conducted by two independent researchers, and the search strategy was primarily designed to identify relevant studies on the effectiveness of CDSSs in improving antibiotic use, as a primary outcome, and on the acceptability and usability of CDSSs in the daily routine of health professionals, patients, and all users of these systems, as a secondary outcome. The following keywords and their equivalents were used in PubMed and EMBASE:

(clinical-decision-support-system OR decision-support-system OR computer-assisted decision-making OR expert-system OR decision-support) AND (antimicrobial resistance OR antimicrobial OR antibiotic* OR antimicrobial management) AND (electronic health OR e-health) NOT Tele-health.

The selection criteria applied in this review were as follows: (1) Language: the papers had to be published in English, Spanish, or Portuguese; (2) Condition or domain being studied: respiratory infections; (3) Type of outcome: studies had to describe the impact of e-health tools on antibiotic use; (4) Participants/population: health professionals and patients; (5) Types of study to be included: randomized and non-randomized (including cluster) trials and observational (including case-control, cross-sectional, cohort, before and after, and interrupted time series) studies. Study protocols, reviews, systematic reviews, and meta-analyses were excluded.

All titles resulting from the database searches were independently reviewed. The inclusion and exclusion criteria were applied by two independent researchers (E.C., M.E.), and subsequently validated by a third researcher (T.H.) in cases where there was no agreement.

### 2.3. Quality Assessment of the Included Studies 

The quality of the included studies was assessed using a scale based on Garg’s study, which comprises judgement and support for the judgement of each included study [23,24]. For each study, risk of bias and quality assessments were conducted separately by two researchers (EC, ME). In cases of disagreement, a third reviewer acted as a referee in order to reach a consensus (TH). The quality assessment was conducted based on five main characteristics, scored from 0–2:

(1) Allocation of study groups (random: 2, quasi-random: 1, selected controls: 0);
(2) Unit of allocation (cluster (such as a practice): 2, physician: 1, patient: 0);
(3) Baseline differences (presence of baseline differences with statistical adjustments: 2, baseline with no adjustments: 1, no baseline differences: 0);
(4) Objectivity of the outcome (blinded assessment: 2, no blinding but defined assessment criteria: 1, no blinding and poorly defined: 0);
(5) Completeness of follow-up (>90%: 2, 80–90%: 1, <80% or not described: 0).

Each study was scored from 0 to 10, based on the sum of the scores for each characteristic. Higher scores represent higher quality studies [23,24].

### 2.4. Data Extraction and Analysis

The analyzed articles were summarized in two tables containing the author’s information, date of publication, country, study design, population, source data, and outcomes, namely, whether the interventions had an impact on antibiotic use or not. The data were extracted independently by two researchers (EC, ME) and their assessments were compared. In cases of disagreement, a third and fourth reviewer (TH, FR) acted as referees in order to reach a consensus.

## 3. Results

### 3.1. Study Selection

**Figure 1:**
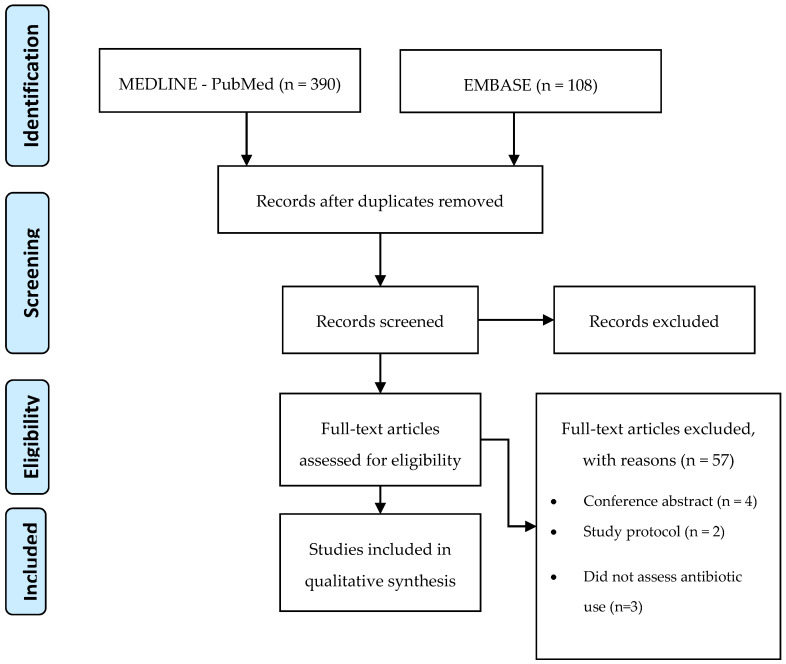
Application of search strategies to retrieve the total number of studies for analysis.

After extracting all 498 articles from the databases, the eligible articles were selected based on the title and abstract. The inclusion criterion of the studies was the impact of the e-health tools on antibiotics use for respiratory tract infections, namely, antibiotics prescription, antibiotics consumption, and adherence to guidelines on antibiotics prescription. All studies that mentioned this impact were considered. After the selection, based on the title, abstract, and review of duplicates, 79 full-text articles were assessed, of which 22 were considered eligible to be included in the present review [25,26,27,28,29,30,31,32,33,34,35,36,37,38,39,40,41,42,43,44,45,46,47] (Figure 1).

### 3.2. Quality Assessment

All of the studies were evaluated regarding their quality. The average score for all the studies was 5.57. Seven studies had a score of 4/10 or below [28,32,35,38,40,43,46]. More than half of the studies (54.5%) had a total score between five and seven [25,26,27,29,30,31,33,34,36,37,39,47]. Three other studies had a score above seven [41,44,45]. The results are presented in Table S1.

### 3.3. Study Characteristics

Information on the study design, location, setting, study population, diseases, and outcomes assessed were retrieved from the included articles. This section sums up the study characteristics of the included papers (Table 1).

#### 3.3.1. Study Design

Of the 22 included studies, 8 (36.3%) were randomized controlled trials, which is the gold standard for intervention effect assessment [25,27,32,33,36,41,44,45]. Six other studies were pre-post studies (27.2%) [29,30,31,40,43,46]. Five studies (22.7%) were retrospective studies [28,34,38,47]. Two observational studies that did not specify the type of study design were also considered [37,39], as well as one study with a mixed-methods design [35].

#### 3.3.2. Location

Most studies (81.8%) took place in the USA [25,26,27,28,29,30,31,32,33,34,36,37,38,39,40,41,46,47]. Three studies (13.6%) were undertaken in the UK [35,44,45], and one was conducted in Australia (4.5%) [43].

#### 3.3.3. Setting

Most of the studies (68.1%) took place in primary care/ambulatory practices [27,28,29,30,31,34,35,36,37,39,41,44,45,46]. Five of the interventions (22.7%) occurred in the context of hospital care [26,38,40,43,47], and 9.0% occurred in academic medical centers [32,33]. The remaining interventions occurred in a pediatric practice [25].

#### 3.3.4. Study Population

Sixteen out of the 22 included studies considered the results obtained based on the entire population [26,28,29,30,32,33,34,35,36,37,38,39,41,43,46,47]. Four studies only measured the adult population [31,40,44,45]. One study only considered children and adolescents [25], and one other study did not assess the impact on the population at all [27].

#### 3.3.5. Diseases

A total of 13 out of the 22 articles assessed antibiotic use related to respiratory tract infections/acute respiratory diseases [25,26,27,28,29,30,31,34,35,38,39,44,45]. Three studies measured the impact of CDSS tools associated with pneumonia [40,43,47]. Three studies considered both streptococcal pharyngitis and pneumonia [32,33], and one article evaluated clinical cases of sinusitis and pharyngitis [46]. Two other studies took into account either sinusitis [37] or uncomplicated acute bronchitis [41].

#### 3.3.6. Intervention

Eight interventions studied CDSSs that were used to aid in the diagnosis of respiratory diseases [25,30,32,33,36,37,41,46], while ten studies focused on the treatment of these diseases [26,28,34,35,38,40,43,44,45,47]. Four other studies covered both diagnosis and treatment [27,29,31,39]. Most of the studies consisted of forms/templates/algorithms that provided a final recommendation based on the information that the health professionals provided to the system [25,26,27,28,29,30,31,32,33,36,39,40,43,46,47]. However, seven studies operated by providing educational material or alerts on clinical practice [34,35,37,38,41,44,45].

#### 3.3.7. Outcomes

The outcomes were classified into two main groups: (1) antibiotics prescription practices, and (2) adherence to guidelines concerning antibiotics prescription. Overall, 14 out of the 22 studies (63.6%) had statistically significant outcomes related to the interventions [26,30,31,32,36,37,38,39,40,41,44,45,46,47]. Five studies did not assess statistical significance [28,29,34,35,43]. Two studies were not statistically significant [27,33]. One study did not obtain statistically significant outcomes from comparing the control group with the intervention group. However, as the e-health tool was not used on all eligible visits to the intervention group, the study showed statistically significant differences in terms of prescription when comparing the visits in which the CDSS was used with the ones in which it was not used [25].

Seventeen studies (77.2%) assessed the impact of e-health tools on antibiotic prescription [25,27,28,29,30,31,32,33,34,35,36,37,40,41,44,45,47], two of which did not have statistically significant results [27,33]. Fifteen studies showed an impact of e-health tools on antibiotic prescription [25,28,29,30,31,32,34,35,36,37,40,41,44,45,47]. Overall, the studies showed positive results on antibiotics prescription of improving the quality or reducing the number of antibiotics prescriptions. However, some heterogeneity in the strength of the effectiveness of CDSSs can also be noted, as some studies show modest, albeit positive and significant, results. One article simultaneously evaluated both the acceptance of the tool used in the intervention and its impact on antibiotic prescription [33]. However, while the tool acceptance was statistically significant, the impact on prescriptions was not [33]. The results are summarized in Table 2.

Five studies (21.7%) evaluated the impact of e-health tools on the adherence to guidelines/prescription congruence and adequacy, out of which four had statistically significant improvements on this outcome [26,38,39,46]. One study did not assess statistical significance, although it had positive results [43]. The results reflect an overall improvement in guideline concordance. Guideline adherence also improved when CDSS tools were used more than once [39]. One study also assessed the impact of CDSS withdrawal, observing an improvement in guideline-discordant antibiotic use, which reinforced the positive impact of CDSSs on guideline adherence [26]. The results are summarized below in Table 3.

Some studies also evaluated the acceptability and/or usability of the tools used in the interventions [25,32,35,39,46]. Overall, clinicians’ perceptions emphasized the usefulness of the systems with positive opinions [25,35]. In Hingorani’s study, the system was used in 40.5% of the visits [46]. In one study, the system scored highly in terms of usability, presenting very positive results [39]. Regarding Mann’s intervention, a heterogeneity in the acceptance of the system components can be observed, with higher acceptance rates towards the lower risk of strep throat or pneumonia diagnoses and lower rates in higher risk diagnoses. When considering diagnoses and antibiotics in combination, only 14% completed the Smartset order [32].

## 4. Discussion

Overall, it appears that e-health tools have a positive impact both on the prescription and conscientious use of antibiotics in relation to respiratory tract infections for prescribing healthcare professionals. However, almost a third of the studies did not present statistically significant results [27,33] or did not assess statistical significance at all [28,29,34,35,43]. Considering all of the 22 included studies, it can be seen that the tools are mainly focused on antibiotics prescription practices, which generally resulted in positive outcomes, whether as a consequence of improving the quality of prescription or reducing the overall number of antibiotics prescriptions. This significantly positive impact on the quality of antibiotics prescription is in agreement with the literature, either in the context of primary [48] or hospital care [49]. CDSS tools have been shown to be effective in improving antibiotics prescription in primary care, and hospitals have been increasingly adopting electronic medical record systems, which have allowed for the emergence of new opportunities in integrating antimicrobial hospital policies, decision support, and antimicrobial usage and surveillance. However, further high-quality research in both contexts should be conducted in order to consistently assess the impact of these tools on clinical practices [48,49].

It is interesting to note that the literature emphasizes that different study designs answer different questions, and researchers should choose the most appropriate study design to evaluate CDSS tools according to their setting [50]. While randomized controlled trials, as well as other experimental designs, are adequate for studying specific changes in clinical practice behaviors, Kaplan et al. [50] argue that they might not suit investigations on other issues, such as the effects associated with whether or not systems are used [50]. Rawson et al. [51] argue that the study designs used to investigate these interventions usually require a standardized view of CDSSs, involving essentially the selection of heterogenous and non-standardized outcomes. These outcomes, namely, the total number of antimicrobial prescriptions, do not directly measure clinical outcomes, such as mortality, adverse events, and the development of antimicrobial resistance, which might constitute a problem in measuring the overall effectiveness of these tools [51].

The selected studies in which the intervention measured antibiotic prescription as the main outcome displayed some discrepancies associated with the significance of their results. In Bourgeois’ study [25], no significant difference was detected in the total antibiotic prescriptions between clinicians in both the intervention and control groups. However, when the authors took into consideration the fact that most of the participants in the intervention group did not use the CDSS tool as expected, it became evident that the ones who used it had significantly reduced the total antibiotic prescriptions [25]. For this reason, user behavior appears to be an important outcome to assess in connection with this type of intervention, since it might have an important impact on the obtained results.

Regarding guideline adherence, e-health tools have been shown to have a positive effect in all considered studies, which is congruent with the literature [17,23]. However, despite these positive results, it is important to note that one of these studies did not assess the statistical significance of guideline concordance [43].

These alterations on clinical practices arising from the increased use of e-health tools, especially regarding antibiotic prescription quantity and quality, may ultimately reduce several problems associated with inadequate antibiotic use, namely, antibiotic resistances. In general, the quality of healthcare constitutes the major facilitator of e-health interventions, while costs are the major barrier [52]. For this reason, researching the implementation of these tools in a clinical and real context, thus allowing for a realistic assessment of their influence on clinical practice, is essential.

Studies highlighting the usability of these tools are emerging, and several scales of the usability of e-health tools have recently been published [53]. These types of studies, as well as acceptability studies, allow for a more profound analysis of the role of e-health tools in a clinical context. It is also very important that studies provide detailed information not only on the intervention’s methodology per se, but also on e-health tools, in order to improve reproducibility and allow for similar research in other contexts. The challenge of designing information systems for a domain as complex as healthcare should be recognized. Few guidelines exist that aim to allow developers to follow common, effective, and safe practices, but significant advances can be achieved by focusing on human factors and a user-centered design, as the tools are built in consideration of the user, instead of forcing the adaptation to an idealized tool [32,54]. Despite the impact that user-centered designs have on the acceptance of e-health tools, only three studies [32,33,36] considered a user-centered design for their e-health tools, which might constitute an obstacle to the optimization of the remaining systems.

Some studies also assessed the usability and acceptance of the e-health tools used in the interventions [25,32]. The user reports given by clinicians were positive, and it is widely believed that these tools can strongly improve clinical practice and aid in the improvement of the quality of antibiotics prescription [25,32,33,35,39]. Health professionals also emphasized the ease of use of these tools. However, it is important to note that each of these e-health tools may have a different learning curve, based on their intrinsic complexity and the overall familiarity that health professionals have with these types of software. Younger physicians also appeared to accept e-health tools more easily than older practitioners. Health professionals with higher levels of training appeared to be less accepting of CDSSs. For this reason, CDSS engagement should be tailored based on age and training level to improve usability and acceptance [33].

Based on Sirajuddin’s study [55], modern CDSSs should adhere to key principles, like the CDS Five Rights model. This model suggests that sustainable improvements are more likely if they communicate “the right information to the right person, in the right format, through the right channel, and at the right time” [55]. Conducting implementation research on this topic, focusing mainly on implementation issues associated with CDSS tools, with the main objective of supporting and promoting successful interventions that have been demonstrated to be effective, is highly important [56]. Despite its relevance, it is a somewhat neglected field of study, either due to a lack of investment in implementation research activities or a lack of overall understanding on what implementation research has to offer. Despite the high investment in health innovation, research that considers how innovative tools can be better used and implemented has not attracted significant funding [56]. This might be the reason why a vast majority of e-health interventions tend to fail clinical implementation, despite displaying promising research results [52]. In order to attain a successful outcome, the assessment of e-health interventions should be based essentially on three pillars of care: access, quality, and cost containment [52].

This study has various strengths, namely, the extensive and systematic research of articles on the stated topic. However, one of the limitations of this paper is its use of only two databases (PubMed and EMBASE), which may have led to a lack of consideration of other potentially relevant articles on other databases. Given the heterogeneity of methods, interventions, and outcomes, a meta-analysis of the effectiveness of the interventions could not be performed. This heterogeneity also brings some barriers to the drawing of some conclusions based solely on the outcomes, considering the differences between the results obtained, for instance, regarding guideline adherence. Another limitation is associated with the timeline of some of these studies, since they are more than ten years old, and at that time, CDSS tools were not as prevalent as they are nowadays.

After the quality assessment, it can be noted that some studies might present an overall higher bias risk. The acquisition of a low average score (5.57) may indicate that studies on the effect of CDSSs on antibiotics have a poorer methodological quality when compared to studies on other drug groups or other types of therapeutic interventions [23,24].

While CDSS tools have an overall potential to be powerful in enhancing clinical care while, at the same time, offering a promising future for optimizing antibiotic prescription, it may be difficult to generalize, as the vast majority of the studies were conducted in the United States, and they may therefore not reflect the diversity of healthcare worldwide regarding clinical practice, prescription behaviors, and even policies on antibiotics use [57].

## 5. Conclusions

This review indicates that interventions using e-health tools, especially CDSSs, can be effective in optimizing and reducing antibiotics prescription. However, it is pertinent to emphasize that the outcomes measured were highly heterogenous and expressed different levels of effectiveness. For this reason, this review only allows for an overall picture of the potential that CDS tools have in relation to antibiotics use. The CDSS interventions themselves were also highly heterogenous, having different approaches concerning antibiotics use, e.g., some tools focused on reducing antibiotic prescriptions, while others turned to guideline adherence or improving the quality of antibiotics prescriptions. Despite these limitations, the included studies revealed that health professionals are very receptive to the use of e-health tools. Antibiotic prescription is a particularly complex area in medical decision-making, so further research is required to determine the characteristics of CDSSs, which are crucial for obtaining a high guideline concordance. The conclusions of this review can be used to enrich the debate on the impact of CDSSs on antibiotic optimization.

## Figures and Tables

**Table 1:** Synthesis of the studies’ characteristics and respective outcomes.

Author (Year)	Title	Study Design	Location	Setting	Disease	Study Population	Intervention
Bourgeois FC (2010) [25]	Impact of a computerized template on antibiotic prescribing for acute respiratory infections in children and adolescents.	RCT	USA	Pediatric practice	RTI	Children and Adolescents	Template for diagnosis with clinical support
Gifford J (2017) [26]	Decision support during electronic prescription to stem antibiotic overuse for acute respiratory infections: a long-term, quasi-experimental study.	Retrospective study	USA	Hospital care	RTI	ALL	CDSS deployed at the moment of AB prescription
Ginzburg R (2018) [37]	Using Clinical Decision Support Within the Electronic Health Record to Reduce Incorrect Prescribing for Acute Sinusitis.	Observational cohort	USA	Primary care clinics	Sinusitis	ALL	Best practice alert
Gonzales R (2013) [41]	A cluster randomized trial of decision support strategies for reducing antibiotic use in acute bronchitis.	CRCT	USA	Primary care clinics	Uncomplicated acute bronchitis	ALL	Best practice alert
Grayson ML (2004) [43]	Impact of an electronic antibiotic advice and approval system on antibiotic prescribing in an Australian teaching hospital.	Prospective, Non-randomized: Pre/post-study	Australia	Hospital care	CAP	ALL	Computer-generated AB approval
Gulliford MC (2014) [44]	Electronic health records for intervention research: a cluster randomized trial to reduce antibiotic prescribing in primary care (eCRT study).	RCT	UK	Primary care clinics	RTI	Adult	CDSS with education and decision support
Gulliford MC (2019) [45]	Effectiveness and safety of electronically delivered prescribing feedback and decision support on antibiotic use for respiratory illness in primary care: REDUCE cluster randomized trial.	CRCT	UK	Primary care clinics	RTI	Adult	Webinar + AB reports + Decision support tools
Hingorani R (2015) [46]	Improving antibiotic adherence in treatment of acute upper respiratory infections: a quality improvement process.	Prospective, Non-randomized: Pre/post-study	USA	Primary care clinics	Sinusitis, pharyngitis	ALL	Didactic teaching, AB guidelines, CDSS integrated on EHR
Jones BE (2018) [47]	In Data We Trust? Comparison of Electronic Versus Manual Abstraction of Antimicrobial Prescribing Quality Metrics for Hospitalized Veterans With Pneumonia.	Retrospective study	USA	Hospital care	Uncomplicated pneumonia	ALL	Electronic *vs* manual Medication Use Evaluation (MUE)
Linder J (2007) [29]	Clinical decision support to improve antibiotic prescribing for acute respiratory infections: results of a pilot study.	Prospective, Non-randomized: Pre/post-study	USA	Primary care clinics	RTI	ALL	ARI Smart Form: assistance in AB prescription for RTI visits
Linder JA (2006) [28]	Acute infections in primary care: accuracy of electronic diagnoses and electronic antibiotic prescribing.	Retrospective study, (double) cross-sectional	USA	Primary care clinics	RTI	ALL	Use of electronic prescribing
Linder JA (2009) [27]	Documentation-based clinical decision support to improve antibiotic prescribing for acute respiratory infections in primary care: a cluster randomized controlled trial.	CRCT	USA	Primary care clinics	RTI	n.m.	ARI Smart Form: assistance in AB prescription for RTI visits
Litvin CB (2013) [30]	Use of an electronic health record clinical decision support tool to improve antibiotic prescribing for acute respiratory infections: the ABX-TRIP study.	Prospective, Non-randomized: Pre/post study	USA	Primary care clinics	RTI	ALL	ABX-TRIP: guidelines, diagnostic criteria, AB use recommendation
Mainous AG (2013) [31]	Impact of a clinical decision support system on antibiotic prescribing for acute respiratory infections in primary care: quasi-experimental trial.	Prospective, Non-randomized: Pre/post-study	USA	Primary care clinics	RTI	Adult	CDSS on EHR, helps with appropriate diagnosis and AB suggestions
Mann D (2014) [32]	Measures of user experience in a streptococcal pharyngitis and pneumonia clinical decision support tools.	RCT	USA	Academic center	streptococcal pharyngitis and pneumonia	ALL	CDSS tool (iCPR) with Smartset (medication bundled-order set)
McCullagh LJ (2014) [33]	User centered clinical decision support tools: adoption across clinician training level.	RCT	USA	Academic medical institution	streptococcal pharyngitis and pneumonia	ALL	CDSS tool (iCPR) with Smartset (medication bundled-order set)
McCullough JM (2014) [34]	Impact of clinical decision support on receipt of antibiotic prescriptions for acute bronchitis and upper respiratory tract infection.	Retrospective study	USA	Primary care clinics	RTI	ALL	CDSS use assessment
McDermott L (2014) [35]	Process evaluation of a point-of-care cluster randomised trial using a computer-delivered intervention to reduce antibiotic prescribing in primary care.	Mixed methods	UK	Primary care clinics	RTI	ALL	Computer point-of-care
McGinn TG (2013) [36]	Efficacy of an evidence-based clinical decision support in primary care practices: a randomized clinical trial.	RCT	USA	Primary care clinics	Streptococcal pharyngitis and pneumonia.	ALL	Clinical prediction tool
Rattinger GB (2012) [38]	A sustainable strategy to prevent misuse of antibiotics for acute respiratory infections.	Retrospective study	USA	Hospital care	RTI	ALL	CDSS with treatment paths for fluoroquinolones and azithromycin
Rubin MA (2006) [39]	Use of a personal digital assistant for managing antibiotic prescribing for outpatient respiratory tract infections in rural communities.	Observational randomized study	USA	Primary care clinics	RTI	ALL	CDSS with diagnostic and therapeutic recommendation
Webb BJ (2019) [40]	Antibiotic Use and Outcomes After Implementation of the Drug Resistance in Pneumonia Score in ED Patients With Community-Onset Pneumonia.	Prospective, Non-randomized: Pre/post-study	USA	Hospital care	Pneumonia	Adult	DRIP score calculator

AB—Antibiotic; ARI—Acute Respiratory Infection; CAP—Community-acquired pneumonia; CDSS—Clinical decision support system; DRIP—Drug-Resistance in Pneumonia; EHR—Electronic health records; RTI—Respiratory tract infections; (C)RCT—(Cluster) Randomized controlled trial; n.m.—not mentioned.

**Table 2:** Summary of the included studies’ results on antibiotic prescription.

Author (Year)	Population (n)	Results	*p*-Value/CI	Observations
Bourgeois FC (2010) [25]	C = 12, P = 146, V = 419	(1) Intervention group *vs* control group: 39.7% *vs* 46% prescription rate; * (2) Intervention group: with ARI-IT users *vs* non-ARI-IT users: 31.7% *vs* 39.9% prescription rate.	(1) *p* = 0.844; * (2) *p* = 0.02	Usability: ARI-IT likely to improve efficiency
Ginzburg R (2018) [37]	P= 54, V = 438	(1) Prescription reduction: 86.3% to 61.7%; (2) Incorrect prescription: 88.5% to 78.7%.	(1) *p* < 0.01; (2) *p* = 0.02	
Gonzales R (2013) [41]	C = 12, P = 155, V = 12826	Prescription reduction: 74.3% to 60.7%	*p* = 0.014	
Gulliford MC (2014) [44]	C = 100, V = 603 409	Prescription reduction by 9.69%.	*p* = 0.034	
Gulliford MC (2019) [45]	C = 79	Prescription intervention group *vs* control group (RR = 0.88).	CI (0.78–0.99); *p* = 0.040	No effect in children < 15 years and adults > 84 years
Jones BE (2018) [47]	C = 30, P = 111, V = 2004	Evaluations as excessive AB duration: mMUE = 82.3%, eMUE = 84.0%	*p* < 0.001	
Linder J (2007) [29]	P = 10, V = 26	Prescription reduction: Intervention group = 35% *vs* control group = 38%,	-	
Linder JA (2006) [28]	C = 9, P = 96	AB prescription on 45% of ARI visits	-	Electronic prescription increased from 2000 (15%) to 2003 (25%) (*p* = 0.03), becoming non-significant after clustering by clinic (*p* = 0.18) or clinician (*p* = 0.23)
Linder JA (2009) [27]	C = 27, P = 443, V = 21961	Prescription rate: Intervention group = 39% *vs* control group = 43% (OR = 0.8) *	CI (0.5–1.3) *	
Litvin CB (2013) [30]	C = 9, Ph=27, N = 6, A = 6	(1) Inappropriate AB use: +1.57% *, (2) Broad spectrum AB use: −16.30%	(1) CI (−5.35%, 8.49%) *; (2) CI (−24.81%, −7.79%)	
Mainous AG (2013) [31]	C = 70	(1) Inappropriate AB use: Intervention group *vs* control group: −0.6%/+4.2%/; (2) Broad-spectrum AB use: Intervention group *vs* control group: −16.6%/+1.2%	(1) *p* = 0.03; (2) *p* < 0.0001	
Mann D (2014) [32]	P = 168, V = 586	Reduced prescription using Smartset (OR = 0.5)	CI (0.3–0.9); *p* = 0.01	Acceptance of iCRP components (diagnosis and antibiotic combination: 14%)
McCullagh LJ (2014) [33]	P = 168, V = 556	Antibiotics ordered using Smartset: PGY1 = 26.4%, PGY2 = 24.3%, PGY3 = 33.1%, Attendings = 37.1%	*p* = 0.52	
McCullough JM (2014) [34]	V = 3317	Use of CDSS associated with a 19% lower likelihood of prescription	-	
McDermott L (2014) [35]	C = 100, P = 103	System could decrease AB prescription rates	-	Useful features of CDSS
McGinn TG (2013) [36]	V = 984	AB prescription: intervention group *vs* control group (RR = 0.74)	CI (0.60–0.92)	
Webb BJ (2019) [40]	V = 2169	Broad-spectrum antibiotic use (OR = 0.62)	CI (0.39–0.98), *p* = 0.039	

C—Clinics/Practices; P—Providers; V—Visits/Cases/Patients; Ph—Physicians; N—Nurses; AB—Antibiotic; EHR—Electronic health record; CDSS—Clinical decision support system; A—Physician Assistants; PGY—Post-graduate year; MUE—Medical use evaluation; ARI-IT—Acute Respiratory Illness Interactive Template; OR—Odds ratio; RR—Risk ratio; CI—Confidence interval; *—not statistically significant.

**Table 3:** Summary of the included studies’ results on guideline concordance/adherence.

Author (Year)	Population (n)	Results	*p*-Value/CI	Observations
Gifford J (2017) [26]	V = 1131	Adjusted odds of guideline concordance *vs* “all other antibiotics”: - Azithromycin (OR = 8.8), - Gatifloxacin (OR = 24.4), - Fluoroquinolone (OR = 5.5)	CI Az (5.7–13.6); CI GT (9.0–66.3); CI Fl (CI 3.5–8.8)	
Grayson ML (2004) [43]	V = 2000	Exact concordance/concordance in 76% of the cases	-	
Hingorani R (2015) [46]	Ph = 27, N = 1, V = 240	Intervention group = 91.25% *vs* control group = 78.6%	*p* < 0.001	Usage rate: 40.5%
Rattinger GB (2012) [38]	V = 3831	Congruent prescription (RR = 2.57)	CI (1.865–3.540)	
Rubin MA (2006) [39]	V = 14393	82% adherence to CDSS, 2.7% change	*p* = 0.016	Usability score of 4.6 (on a 1–5 scale)

C—Clinics/Practices; P—Providers; V—Visits/Cases/Patients; Ph—Physicians; N—Nurses; AB—Antibiotic; OR—Odds ratio; RR—Risk ratio; CI—Confidence interval.

## Supplementary Materials

The following are available online at https://www.mdpi.com/2079-6382/9/8/505/s1, Table S1: Quality assessment results.
