# Supplementary material for: E-Health Tools to Improve Antibiotic Use and Resistances: A Systematic Review
Source: Antibiotics (Basel). 2020 Aug 12;9(8):505. doi: 10.3390/antibiotics9080505 (PMC7460242; doi:10.3390/antibiotics9080505)
Supplement: Supplementary file 1 [file antibiotics-09-00505-s001.pdf]

Supplementary Material

Table S1. Quality assessment results.

Author (year)	Allocation of Study Groups	Unit of Allocation	Baseline Differences	Objectivity of the Outcome	Completeness of Follow-up	Total Score
**Bourgeois FC (2010)**	2	2	2	1	0	**7**
**Gifford J (2017)**	2	0	2	1	2	**7**
**Ginzburg R (2018)**	0	1	2	2	0	**5**
**Gonzales R (2013)**	2	2	2	2	2	**10**
**Grayson ML (2004)**	0	0	0	1	0	**1**
**Gulliford MC (2014)**	2	2	2	2	2	**10**
**Gulliford MC (2019)**	2	2	2	2	2	**10**
**Hingorani R (2015)**	0	0	1	1	0	**2**
**Jones BE (2018)**	2	2	0	1	0	**5**
**Linder J (2007)**	0	1	0	2	2	**5**
**Linder JA (2006)**	0	0	0	2	0	**2**
**Linder JA (2009)**	2	2	1	1	0	**6**
**Litvin CB (2013)**	0	2	0	2	2	**6**
**Mainous AG (2013)**	0	2	2	1	0	**5**
**Mann D (2014)**	2	1	0	1	0	**4**
**McCullagh LJ (2014)**	2	1	1	1	1	**6**
**McCullough JM (2014)**	2	1	2	1	0	**6**
**McDermott L (2014)**	0	1	0	1	1	**3**
**McGinn TG (2013)**	2	1	2	1	0	**6**
**Rattinger GB (2012)**	0	0	1	1	0	**2**
**Rubin MA (2006)**	2	2	2	1	0	**7**
**Webb BJ (2019)**	0	0	2	1	0	**3**
